# Epidemiological Data on Heart Rhythm Disorders in India - A Formidable but Surmountable Challenge

**DOI:** 10.1016/s0972-6292(16)30478-8

**Published:** 2012-04-30

**Authors:** B Hygriv Rao

**Affiliations:** Senior Consultant Cardiologist and Electrophysiologist, KIMS Hospitals, Secunderabad

**Keywords:** Heart Rhythm Disorders, Epidemiology, India

The arena of cardiovascular diseases in the world has consistently been associated with paucity of contribution of data from India. There are currently serious and focused attempts at acquiring and analyzing global data on heart rhythm disorders, particularly the problems of heart failure, atrial fibrillation and sudden death. The compilation of global statistics is bound to be incomplete without representation of figures and trends from a country of over a billion constituting 1/6th of the world population. The challenges associated with data collection are real and result from unique situations prevailing in this part of the world [1,2]. There is a serious impediment to assessment of mortality figures as there is a gross under registration of deaths with only a small percentage of them being medically certified. Even when certified there is uncertainty in the reliability of death certificates due to inconsistent physician attribution of causes, absence of uniform codes and standardization in the data entered in the certificates. The information obtained from first responders is limited as there are no system-wide emergency services and is accessible to small minority of the population in select urban communities and autopsies in non-medico legal cases are practically nonexistent. Furthermore, for the majority of the population including patients with cardiovascular diseases periodic physician visits are infrequent and out patient medical records are seldom available or feasible for analysis. Incidence and prevalence of episodic clinical events like atrial fibrillation are hence difficult to obtain. Absence of adoption of uniform and standardized coding by hospitals for classification of diseases imposes restriction on identification of morbidities like heart failure. Contact details of patients not infrequently are erroneous, not updated or incomplete making it difficult for investigators to collect follow up data.

These facts doubtless indicate futility of employing conventional epidemiological tools in data collection in India. Inability to utilize these resources should not however be a deterrent to our efforts to capture and contribute data from this part of the world. We should explore alternative methods which are more feasible and practical in our population. Verbal autopsy has been shown to be a viable strategy in assessment of various components of mortality and has been effectively used to achieve this objective in large rural studies [3,4]. It has also been incorporated in surveillance studies to assess cardiovascular mortality and introduce effective preventive measures [5]. Using standardized questionnaires, PURE study provided very insightful data on the large variations in compliance with secondary prevention medications between developed and third world countries [6]. No doubt, these studies are difficult to design, rather cumbersome to conduct, are human resource intensive and have been criticized [7]. The positive attributes of these studies are they can be done at a modest cost and generate data in situations where it would otherwise be very difficult. Innovativeness and flexibility in tailoring the design of the questionnaires to specific study and target specific segments of the population improves the accuracy and reliability of the data. Such a custom designed methodology was used to obtain burden of sudden cardiac death in the community in the state of Andhra Pradesh [8]. Review and rechecking the data at more than one level and adjudication of inferences by multiple independent physicians improves the validity of the studies. The importance of indigenous data cannot be undermined as it influences the therapeutic protocols followed by the physician community and helps prioritizing health care resources at national level. The results of the few studies available clearly document the fact that cardiac morbidities and mortality occur at a younger age in India. Tasks ahead of cardiologist-investigators are to unravel the geographically relevant factors influencing sudden cardiac deaths, analyze the profiles of atrial fibrillation and their cardiovascular outcomes, and explore the heterogeneity of heart failure population to identify the treatable subsets. The epidemiological research in our country should endeavor to contribute accurate and reliable information pertaining to heart rhythm disorders by unhesitatingly employing methodologies which may be nontraditional but are eminently capable of achieving set objectives under the prevailing adverse medico-social circumstances.
